# Multivariate classification of neuroimaging data with nested subclasses: Biased accuracy and implications for hypothesis testing

**DOI:** 10.1371/journal.pcbi.1006486

**Published:** 2018-09-27

**Authors:** Hamidreza Jamalabadi, Sarah Alizadeh, Monika Schönauer, Christian Leibold, Steffen Gais

**Affiliations:** 1 Medical Psychology and Behavioral Neurobiology, University of Tübingen, Tübingen, Germany; 2 Bernstein Center for Computational Neuroscience, Ludwig-Maximilians-Universität München, Planegg-Martinsried, Germany; 3 IMPRS for Cognitive and Systems Neuroscience, University of Tübingen, Tübingen, Germany; 4 Department of Psychiatry, Division for Translational Psychiatry, University of Tübingen, Tübingen, Germany; 5 Department of Psychology, Ludwig-Maximilians-Universität München, München, Germany; 6 Department of Biology II, Ludwig-Maximilians-Universität München, Planegg-Martinsried, Germany; Oxford University, UNITED KINGDOM

## Abstract

Biological data sets are typically characterized by high dimensionality and low effect sizes. A powerful method for detecting systematic differences between experimental conditions in such multivariate data sets is multivariate pattern analysis (MVPA), particularly pattern classification. However, in virtually all applications, data from the classes that correspond to the conditions of interest are not homogeneous but contain subclasses. Such subclasses can for example arise from individual subjects that contribute multiple data points, or from correlations of items within classes. We show here that in multivariate data that have subclasses nested within its class structure, these subclasses introduce systematic information that improves classifiability beyond what is expected by the size of the class difference. We analytically prove that this subclass bias systematically inflates correct classification rates (CCRs) of linear classifiers depending on the number of subclasses as well as on the portion of variance induced by the subclasses. In simulations, we demonstrate that subclass bias is highest when between-class effect size is low and subclass variance high. This bias can be reduced by increasing the total number of subclasses. However, we can account for the subclass bias by using permutation tests that explicitly consider the subclass structure of the data. We illustrate our result in several experiments that recorded human EEG activity, demonstrating that parametric statistical tests as well as typical trial-wise permutation fail to determine significance of classification outcomes correctly.

## Author summary

When data are analyzed using multivariate pattern classification, any systematic similarities between subsets of trials (e.g. shared physical properties among a subgroup of stimuli, trials belonging to the same session or subject, etc.) form distinct nested subclasses within each class. Pattern classification is sensitive to this kind of structure in the data and uses such groupings to increase classification accuracies even when data from both conditions are sampled from the same distribution, i.e. the null hypothesis is true. Here, we show that the bias is higher for larger subclass variances and that it is directly related to the number of subclasses and the intraclass correlation (ICC). Because the increased classification accuracy in such data sets is not based on class differences, the null distribution should be adjusted to account for this type of bias. To do so, we propose to use blocked permutation testing on subclass levels and show that it can confine the false positive rate to the predefined α-levels.

## Introduction

Multivariate pattern analysis (MVPA) combined with cross-validation and permutation testing allows the use of machine learning algorithms to detect differences between classes of data for statistical hypothesis testing and is frequently used in neuroscience [1–3] and bioinformatics [4–6]. Whereas classical statistical approaches search for individual features in a data set that allow to distinguish two experimental conditions, MVPA analyzes data sets as a whole, searching for distinguishing multi-dimensional patterns. Therefore, it can provide increased sensitivity compared to classical multiple-univariate testing methods in high-dimensional data sets [7–9]. In a typical application of MVPA, a classifier, e.g. a linear support vector machine (SVM), is trained to distinguish different classes (e.g. different experimental conditions, different groups of patients, etc.) on one part of a data set. Then, the classifier is tested on the remaining data. This results in a certain percentage of accurate classifications (correct classification rate [CCR]). To improve accuracy of CCR estimation, a cross-validation procedure is used, which assures that all parts of the data are used for training as well as testing on repeated iterations of the analysis. If the CCR lies significantly above the level expected by chance (e.g. 50% for a two-class problem), it can be concluded that a difference between classes exists.

In the context of classical hypothesis testing, univariate hypotheses are usually phrased in terms of differences of mean values. In a multivariate context, this translates to differences in class centroids. However, using MVPA for hypothesis testing can have a specific vulnerability compared with classical univariate methods if the data have a class-unrelated substructure. Elements that form a subclass (e.g. repeated stimuli in an experiment) share features that are specific to the subclass but not to the class. In univariate statistics, these features average out and contribute to the normal distribution of values. They therefore affect results only minimally. In a multidimensional space, however, subclasses can form distinguishable clusters [10, 11]. A classifier can learn to separate one or several of these clusters based on their distinguishing features instead of on those features shared by all elements of the class. This impedes the use of classifiers for testing general hypotheses regarding class differences.

**Fig 1:**
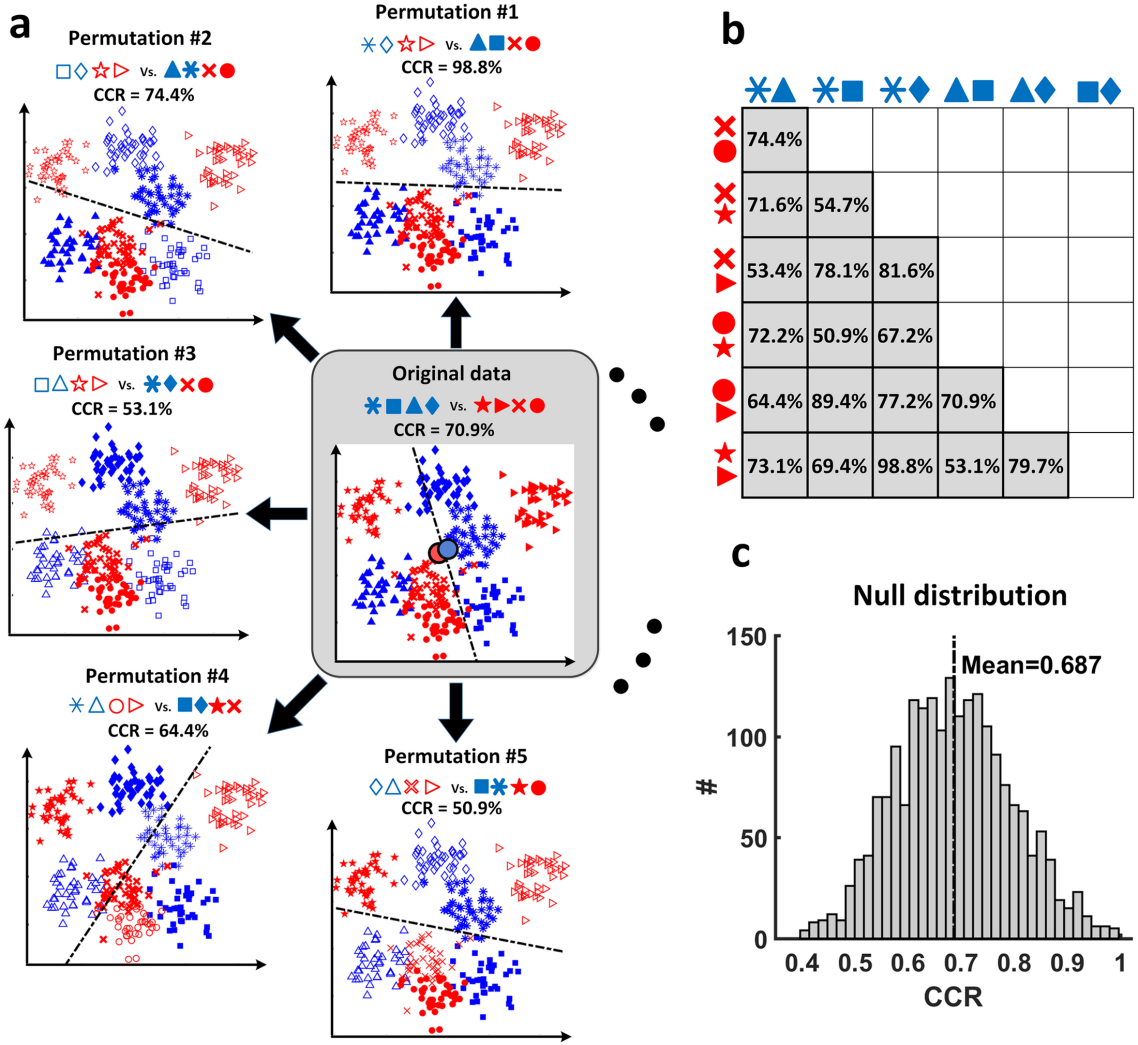
Classification accuracy in data with subclasses can exceed chance level even if data are randomly attributed to two conditions. (a) Center: An exemplary data set with 4 subclasses per class (blue and red). Although classes have almost identical multivariate means (represented by two filled circles in the center), classification of this data set with LDA using 2-fold cross-validation leads to 70.9% classification accuracy. Surrounding plots show five possible random assignments of subclasses to two new classes (“closed-symbol” versus “open-symbol”), if each new class has two subclasses from original classes (blue and red). (b) The data set in the center of (a) can be randomly divided into two new “closed-symbol” versus “open-symbol” classes of 4 subclasses in 18 ways (12(42)2). The table shows all 18 possible configurations with their respective CCRs. Each cell in the table shows the four random subclasses assigned to the newly built “closed-symbol” class (the remaining symbols will belong to the “open-symbol” class) and their corresponding classification accuracy. Note that only few of these random assignments show close to chance level CCRs. The average CCR for all 18 possible randomizations is 71.1%. (c) Simulating 2000 data sets with the same structure as in (a), i.e. two classes, each with four subclasses, identical centroids and variances, results in a null distribution with a mean CCR of 68.7%. Since these data sets are sampled from identical distributions, these classification accuracies represent the empirical null distribution for the data set shown in the center of (a).

In particular, when different subclasses are nested within the classes, the obtained classification accuracies can be systematically higher than the expected chance level, even when data of both classes are sampled from the same distribution, i.e. the null hypothesis is true. This is especially problematic because the goal of MVPA is to extract class-related structure from the data and to better understand underlying mechanisms rather than to enhance classification accuracy per se [2, 12]. A simplified two-dimensional example of a data set with nested subclasses is illustrated in Fig 1. Here, classes A and B each contain four distinguishable nested subclasses. The average CCR is above 50% (here: 70.9%, Fig 1a) although no systematic differences between classes A and B exist, i.e. centroids and variances of class A and B are identical. Without the presence of subclasses, the expected value of the CCR should be 50% for a two-class linear classification [2]. Importantly however, the simple presence of structure due to the subclasses, which is completely unrelated to the classes themselves, allows higher classification rates (Fig 1b and 1c). In this example, separability of eight subclasses along two feature dimensions leads to an average CCR of 71.1% over all possible random attributions of subclasses to classes A and B (Fig 1b). As we will show below, this behavior can be observed to a varying degree in every data set in which classes consist of distinct subclasses (e.g. types of stimuli, groups of subjects, multiple recording sessions, blocks of fMRI recording, temporally correlated trials, etc.). We will present simulations as well as analytical results that show that MVPA with linear classifiers systematically produces inflated accuracies when applied to data containing nested subclasses.

There are three sources of variance that are of interest in the present considerations [13], which describe a multilevel model that can be specified as follows: *y*_*ijk*_ = *C*_*i*_ + *S*_*ij*_ + *ϵ*_*ijk*_. *y*_*ijk*_ are individual measurements, e.g. physiological brain responses to certain stimuli. *C*_*i*∈[1,2]_ represents the class centroids, e.g. the influence of an experimental manipulation, the difference between patient and control group, or the responses to different conditions. *S*_*ij*_ are the centroids of the *j*^*th*^ subclass within class *i* and represent the vector from the class centroid to the subclass centroid. The variance $\sigma_{S}^{2}$ of the set of centroids reflects differences that are class-unrelated [14, 15]. *ϵ*_*ijk*_ reflects the deviation from the subclass mean arising from the within-class error variance $\sigma_{W}^{2}$ and represents e.g. measurement noise. In classical statistics, it has been demonstrated that failing to accommodate for the effect of non-zero subclass variance can produce large false positive rates [16] and the ratio of subclass-to-trial-variance, defined as the intraclass correlation ($ICC\;=\;\frac{\sigma_{S}^{2}}{\sigma_{S}^{2}+\;\sigma_{W}^{2}}$), determines the extent to which subclass variation affects statistical conclusions [16]. Because it is usually difficult to separate the influence of subclasses from the main class effects [17, 18], experiments and statistical analyses must be designed to avoid these confounds. In the present paper, we will investigate the boundary conditions and consequences of this phenomenon when using linear classifiers and describe a method to circumvent false positive results.

## Results

### Practical Example 1: Biased accuracy in hierarchically nested subclasses

The following example illustrates how a nested factor can influence the expected classification accuracy in an experiment that investigates the EEG responses to the visual presentation of digits and letters [10]. The experiment was performed by 19 healthy subjects. All participants were right-handed, between 18 and 30 years old, native German speakers and non-smokers. Subjects underwent EEG recording in two different sessions at two different days while performing a short-term memory task with digits from 0 to 9 and 10 consonant letters, which were selected randomly but remained the same for all the subjects. During each trial of the encoding phase, participants were instructed to memorize strings of 7 digits or 7 letters that were presented sequentially. Each stimulus was shown on a black screen for 100 ms with an inter-stimulus interval of 1 s. After a 4-s maintenance interval, a probe item was presented, and subjects were asked if it had been in the sequence of stimuli. For each stimulus, 18 presentations were used. EEG was recorded using an active 128-channel Ag/AgCl-electrode system (ActiCap, Brain products, Gilching, Germany) with 1 kHz sampling frequency and a high-pass filter of 0.1 Hz. Electrodes were placed according to the extended international 10–20 electrode system.

Here we were interested in whether digits and letters could be distinguished based on the encoding phase of event-related potentials (ERPs). EEG data were low-pass filtered offline at 40 Hz and divided into epochs of one second, starting 50 ms before to 950 ms post stimulus onset. Artefact rejection was done in a semiautomatic process using custom MATLAB scripts based on overall power, extreme amplitude changes, and muscle artefacts. Artefact thresholds were automatically detected based on the variance of the data and manually confirmed upon visual inspection of parameter distributions and of the raw data. Epochs containing artefacts were removed from the data set, channels that contained too many epochs with artefacts were removed and spherically interpolated using routines provided by EEGLAB [19].

To decode brain activity, we classified single-trial EEG using a linear support vector machine (SVM) with 2-fold cross-validation. A 2-fold cross-validation was used, because the resulting classification accuracies have the same mean as other cross-validation schemes but a lower variance. When testing for significance against a null distribution obtained with randomization tests, 2-fold cross-validation therefore has a higher statistical power [2]. As input to the classifier, we used the 1-s ERP response of all 128 channels. The classifier was trained and tested within each subject and session. This procedure resulted in a mean classification accuracy of 54.2% over all subjects and sessions. Using trial-wise permutation with 1000 random repetitions to determine the null distribution, 16 out of 38 sessions had classification accuracies significantly above 50% (p < 0.05). Combined over all sessions [3] the group level 90% confidence interval [CI] is [49.3%, 50.7%].

**Fig 2:**
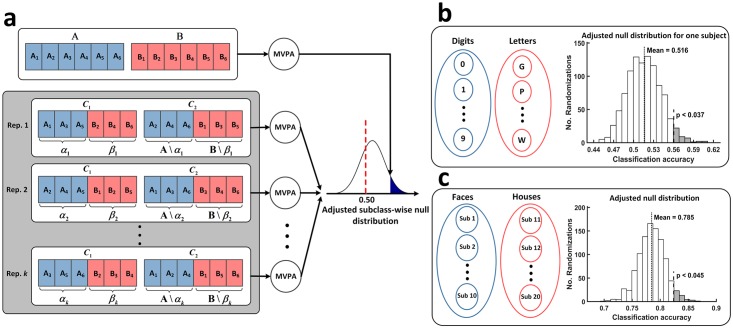
Nested subclass structure biases classification accuracy. This bias can be observed using a block permutation strategy. (a) The flowchart of adjusted permutation test for an exemplary data set with 6 subclasses per class. To cancel the main effect, we build two new classes of *α* and *β* each containing exactly half of the subclasses from A (A_1_, A_2_, …) and B (B_1_, B_2_, …). *A*\*α*_*h*_ and *B*\*β*_*h*_ show the complement of *α*_*h*_ and *β*_*h*_. We build the null distribution by classifying data from newly built classes of *C*_1_ and *C*_2_. The classification accuracy of *A* versus *B* is then compared to this distribution. Importantly, since this procedure preserves the subclass effect, the mean of adjusted null distribution lies beyond 50% if data subclass variance is greater than zero (see Fig 3 for details). (b) Data from Practical Example 1 show subclasses formed by trials belonging to distinct digits and letters. The histogram shows the null distribution of CCRs and the p-value for one subject. (c) Data from Practical Example 2 show subclasses formed by trials belonging to different subjects. The histogram shows the null distribution of CCRs and the p-value.

However, the example in Fig 1 suggests that the classifier might detect only local features of the distinct stimuli (10 digits, 10 letters), which represent 20 subclasses, instead of a generalizable difference between digits and letters. The significant findings in this case could not be interpreted in the sense that the EEG reflects a systematic difference in brain processing of digits and letters, but only in the sense that at least one of the stimuli evokes a distinctive ERP signal. In case both subclass and class effects are present, the CCR will be higher than it could have been expected if only a class effect was present. To quantify this bias, we determined the null distribution of classification accuracies for data with intact subclass structure, but without information about the main classes. To do so, data is permuted in a way that keeps subclasses together but still assigns random class labels, thus effectively removing any class-related information (Fig 2a). This procedure uses permutation at the subclass level instead of the usual trial-level randomization [10, 20]. Trial-level permutation treats every trial as an independent observation and removes class-related as well as other structure from the data. This will result in the null distribution of the data assuming that no systematic relation between trials exists. However, if the data contain subclasses, trials within these subclasses are systematically related. To control for the influence of this structure at the subclass level, the dependencies at the subclass level must be kept intact while removing class-level information [20].

To determine the null distribution, we assign 5 digits and 5 letters to one class and the other 5 digits and 5 letters to the other class. We can draw randomly from 12(105)2=31752 possible permutations of these random assignments if labels of subclasses are distributed between classes in a balanced fashion. Classification results above 50% on average must be related to the subclass structure of the data because class information has been removed. Here, classification over 1000 random permutations results in an average CCR of 50.9% for the two sessions, which deviates significantly from 50% (90% CI: [50.2%, 51.6%]), showing that the subclass variance results in a significant bias (Fig 2b). Using the subject-wise adjusted null distributions, 11 out of 38 sessions remain significantly above chance (*p* < 0.05) compared to 16 sessions without adjustment, showing that using a trial-wise permutation test increases false positive findings considerably.

### Practical Example 2: Subject variability as a nested factor

In another data set with nested subclasses, we analyzed ERP responses to the presentation of pictures of faces and houses, a paradigm that is widely accepted to activate distinct brain regions. EEG was recorded from 20 healthy subjects in one session. The procedure was similar to the experiment in Practical Example 1 with the only difference that the working memory task was performed with sequences of 8 pictures of either faces or houses that were randomly selected from a pool of 100 pictures of faces and 100 pictures of houses. EEG recording and preprocessing was done with the same procedure as in Practical Example 1 above. Our aim here was to test whether pictures of faces and houses elicit distinct ERP responses that can be generalized over subjects. We used artefact-free ERPs from 30 presentations of faces to 10 subjects and from 30 presentations of houses to 10 different subjects, resulting in 300 trials per class. We classified the data with linear SVM using 2-fold cross validation resulting in a classification accuracy of 82.4% (see Fig 2c). Because variability of EEG between subjects is larger than the variability across conditions, EEG trials from distinct subjects establish subclasses in this data set. Therefore, following the logic in Practical Example 1, it cannot be assumed that the classification accuracy is solely driven by the effect of the main classes (here faces vs houses). To address this problem, we treated data from different subjects as subclasses and randomized the class assignment by assigning EEG of faces from 5 subjects and 5 houses to one class and the other 5 subjects with presentation of faces and 5 subjects with presentation of houses to the other class. Like in Experiment 1, we can draw randomly from 12(105)2=31752 possible permutations of these random assignments if labels of subclasses are distributed between classes in a balanced fashion. Here, classification over 1000 random permutations results in a null distribution with a mean of 78.5% and a 90% CI of [74.6–82.2%]. This indicates that there are enormous differences between EEG of different subjects, which strongly bias classification accuracy. The main effect of faces and houses still reaches significance (p < 0.045).

### Simulating biased classification results in data with nested subclasses

**Fig 3:**
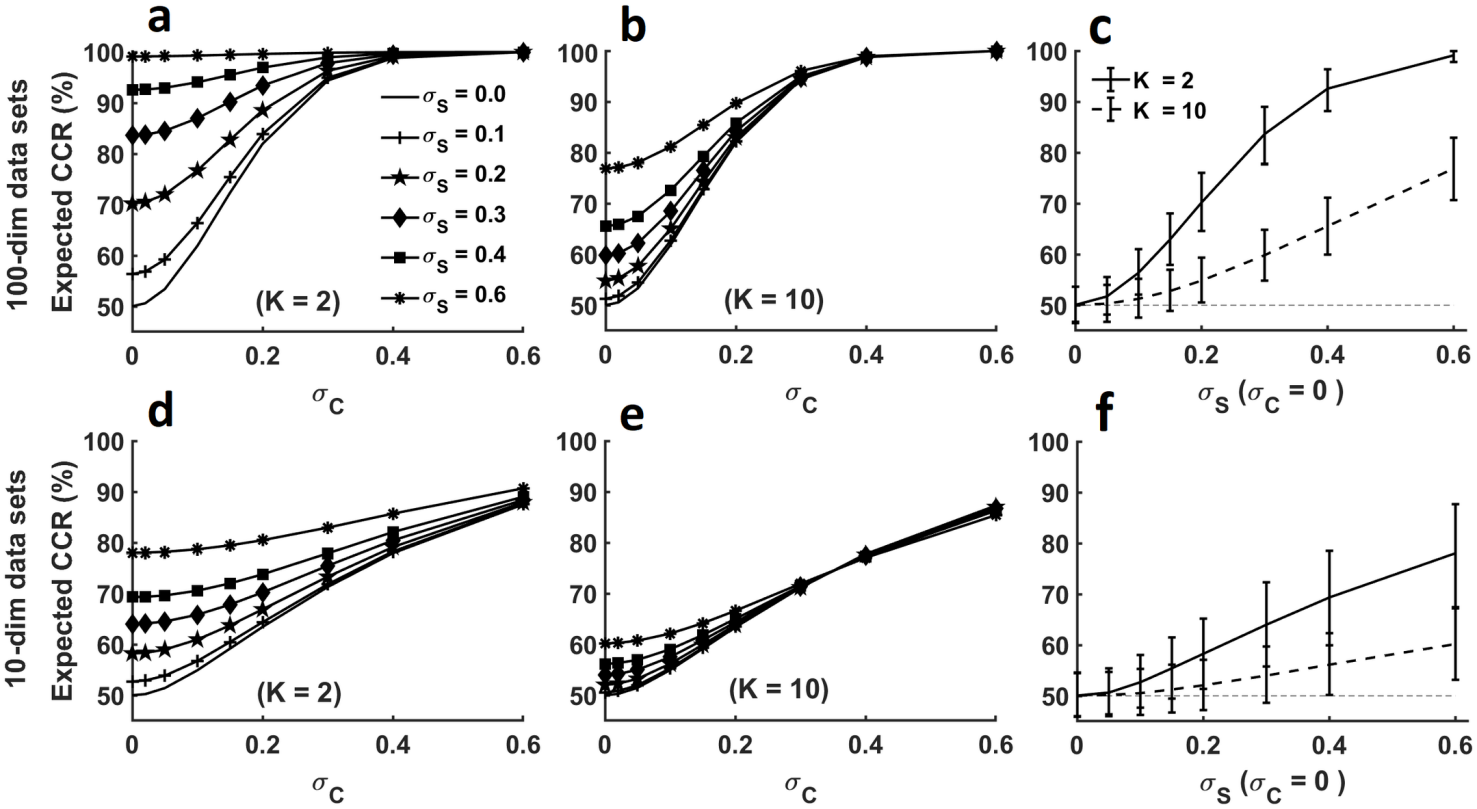
Expected CCRs when data contain subclasses. (a, b) Expected CCRs for 100-dimensional data with a constant trial variance (*σ*_*W*_ = *I*) and varying class and subclass variance ($\sigma_{C}^{2}$ and $\sigma_{S}^{2}$) with *K* = 2 and *K* = 10 subclasses per class. (c) Expected CCRs and standard deviations for 100-dimensional data sets with an effect size of zero (*σ*_*W*_ = 0). (d, e) Expected CCRs for 10-dimensional data with a constant trial variance (*σ*_*W*_ = *I*) and varying class and subclass variance ($\sigma_{C}^{2}$ and $\sigma_{S}^{2}$) with *K* = 2 and *K* = 10 subclasses per class. (f) Expected CCRs and standard deviations for 10-dimensional data sets with an effect size of zero (*σ*_*W*_ = 0).

The previous examples showed that CCRs can in practice diverge from the ground truth chance level. To investigate the effect of nested subclasses on classification accuracies systematically, we used synthetically generated data with varying subclass and class variance according to the multilevel model described in introduction. Nested subclasses are subclasses that do not overlap between the two classes (e.g. 10 letters and 10 digits as in Practical Example 1 above). We studied the distribution of CCRs in four series of two-class experiments where each class contained either 2 or 10 subclasses per class and each observation represented one 10- or 100-dimensional measurement. Each data set consisted of 120 observations per class. Data were sampled from normally distributed populations with identical trial variance (*σ*_*W*_ = *I*) and varying subclass variance (*σ*_*S*_ = *a* × *I*, *a* ∈ [0,0.6]). In addition, we varied the size of the main class-related effect (*σ*_*C*_ = *a* × *I*, *a* ∈ [0,0.6]). We classified data from each simulated experiment with linear SVM (with cost parameter *C* = 1) using 2-fold cross-validation. For each set of parameters, we repeated the whole sampling and classification procedure 5000 times to achieve a stable estimate of CCRs (Fig 3).

If the classes are indistinguishable (i.e. class-related variance $\sigma_{C}^{2}$ is zero, class centroids *C*_1_ = *C*_2_), subclass-effects contribute most strongly to classification accuracy. With increasing between-class variance, the relative influence of subclass variance diminishes (Fig 3a and 3d). A higher number of subclasses also mitigates the influence of the subclass effect (Fig 3b and 3e). From these graphs it is obvious that a high CCR per se does not indicate the presence of class-related information in the data. Rather it is the p-value obtained by comparing the actual CCR with the null distribution of CCRs achieved by removing the class-related variance that should be used as a measure of strength of the main effect. For our simulations, Fig 3c and 3f depict the mean classification accuracies for data sets with no class-related effect but varying levels of subclass-related variance and different numbers of subclasses. It becomes apparent that subclasses bias the CCR expected under the null hypothesis and that this bias is greater in data with fewer subclasses and with more dimensions.

### Theory: Inflated accuracy in data with nested subclasses

The simulations in Fig 3 show that CCRs depend not only on the difference between the classes, but also on subclass variance and on the number of subclasses. To investigate the implications of this observation in more detail, we develop an analytical description of classification rates when data with subclasses are analyzed using linear classifications. We assume our data set to consist of two sets of *N* × *K* independent random vectors ${\vec{x}}_{n}^{\left(k\right)},\;{\vec{y}}_{n'}^{\left(k'\right)}$ where *k*, *k*′ ∈ {1, …, *K*} labels the subclasses and *n*, *n*′ ∈ {1, …, *N*} identifies the sample index in each of the subclasses. The task of the linear classifier is to separate *x* and *y* into two categories. As a model for the linear classifier, we use Linear Discriminant Analysis (LDA). Data distribution within the subclasses is assumed to be Gaussian with variance $\sigma_{W}^{2}$, the distribution of the subclass means is also assumed to be Gaussian with variance $\sigma_{S}^{2}$ and expected values $\hat{\mu },\;\hat{\nu }$. Under these conditions, we determine the expected CCR to be as described in Theorem 1 (Materials and methods, Appendix A). From this theorem directly follows.

Corollary 1: Classification accuracy for data sets with no main class effect ($\hat{\mu }\;=\;\hat{\nu }$) depends only on the number of subclasses and the intraclass correlation *ICC*. It can be estimated by Eq 1.

$$CCR=\;1-\frac{1}{\pi }arctan\left(\sqrt{2\left(\frac{K}{ICC}-1\right)}\right)$$ (1)

Proof: see Materials and methods, Appendix B.

**Fig 4:**
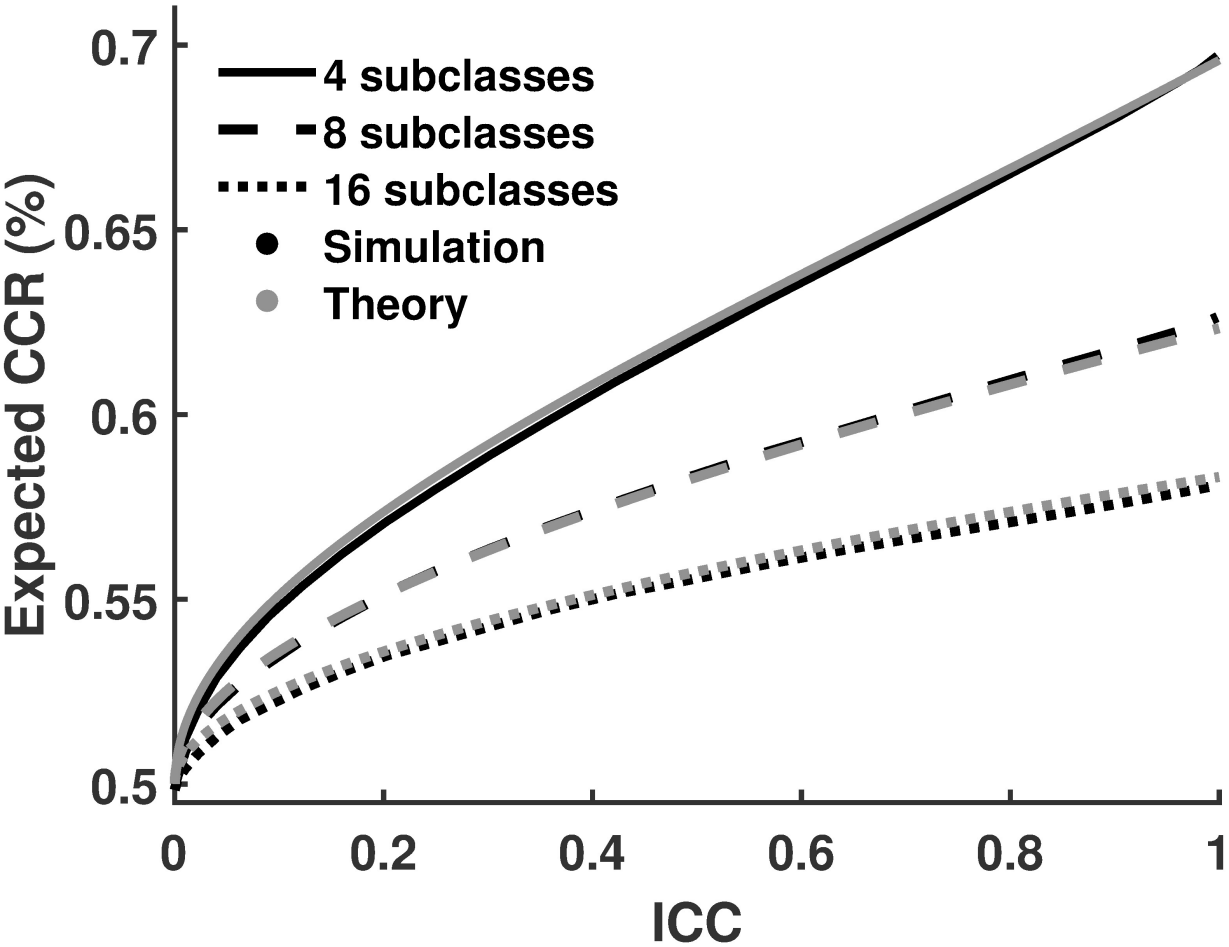
Validation of Formula 1. Expected CCRs for data sets with nested subclasses when the size of main effect is zero once using Eq 1 (gray lines) and once using simulated (black lines). The figure confirms that the analytical solution and simulations produce very similar results.

To validate our analytical results, we generated a series of simulations with a total number of 4, 8, or 16 subclasses and varied *ICC*. We classified these data sets with LDA and compared the CCRs with results of Eq 1. Fig 4 shows that results of the analytical solution and simulations are compatible. According to Corollary 1 (see Fig 4), estimated CCR is a decreasing function of the number of subclasses (*K*) and an increasing function of subclass variance ($\sigma_{S}^{2}$). It also shows that for zero effect size, CCR only depends on the intraclass correlation *ICC* and the number of subclasses *K*. Importantly, CCR is 50% only when *ICC* = 0. It is a monotonically increasing function of *ICC* and of $\sigma_{S}^{2}$.

### Application 1: Biased null distributions to account for inflated accuracy

Since subclass differences inflate classification accuracy, significance tests must take this subclass-related bias into account. To do so, we advocate using a block permutation strategy, which addresses this problem by adjusting the null distribution [20] (see Experiments 1 and 2 for two practical examples). To achieve this, we permute class association on the subclass-level instead of the trial level (see Fig 2 for illustrated examples of such permutation procedures). Specifically, to remove class-related information, we randomly switch class labels of all trials within each subclass to create randomized classes that are balanced in terms of main class effect (see Fig 2a).

The distribution of CCRs for these relabeled data represents the null distribution with which the actual CCR must be compared. Only if classification accuracy for the real data is higher than that for blocked permuted data, it can be concluded that there is a systematic difference between the classes. If too few subclasses exist to generate a sufficient number of random permutations, we propose to use the group null distribution over subjects or sessions, which can be obtained non-parametrically by testing the mean CCR from data over all the subjects against a null distribution that is obtained by repeatedly averaging randomly sampled CCRs from the subclass-level permutations from each subject [3].

### Application 2: Significance bias in data with nested subclasses

**Fig 5:**
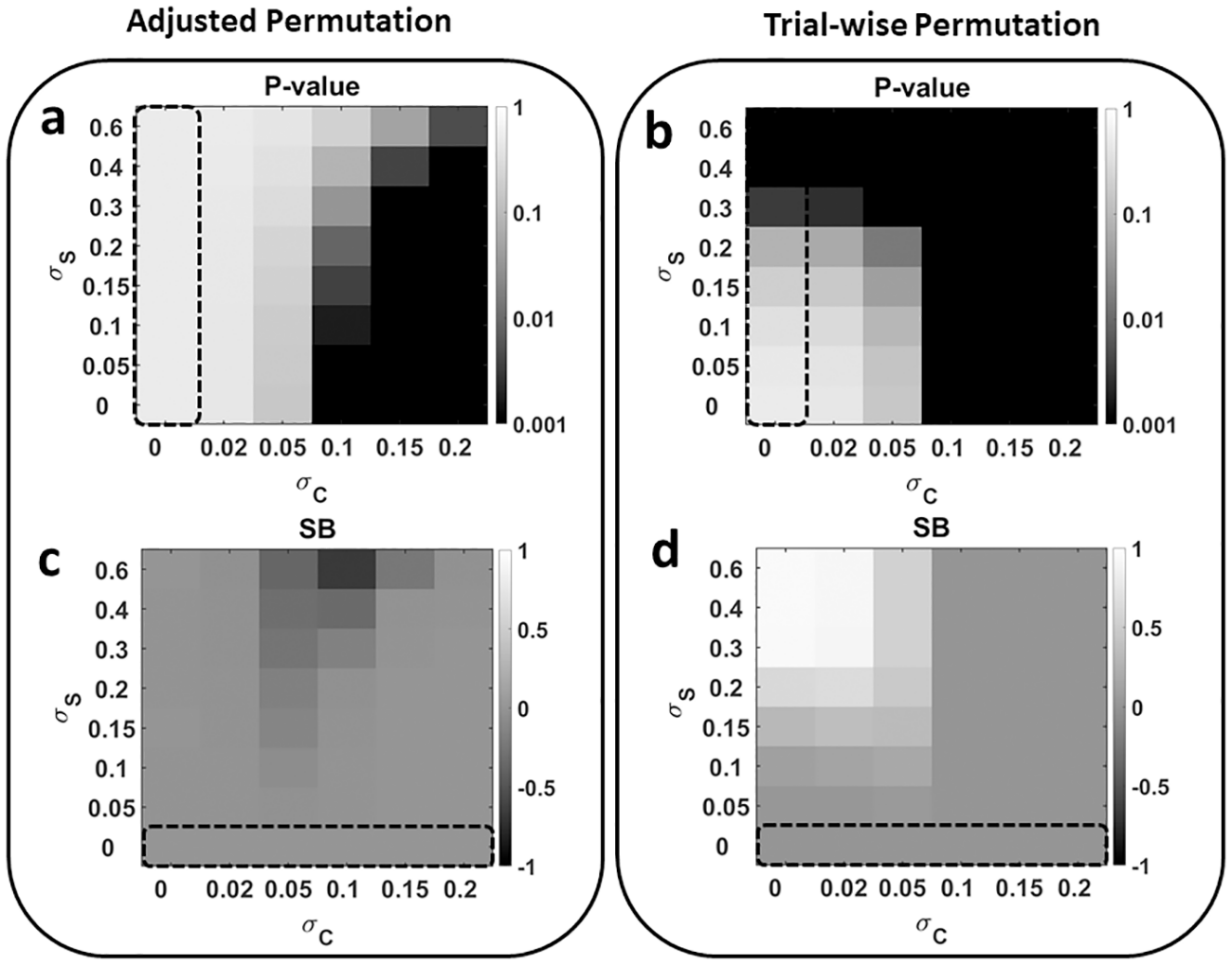
Randomization results for data with subclasses (*K* = 10). (a, b) The area delimited by the dashed rectangle shows the expected p-values for a main effect of zero. Importantly, even when CCRs are strongly biased because of nonzero subclass variance, p-values remain constant for the block-wise randomization test. The trial-wise permutation test fails to remove the bias introduced by subclass variance. Note that even small subclass effects result in falsely positive significance tests (dark grey and black squares for *σ*_*C*_ = 0). In the ideal case, the presence of subclass variance should not affect the percentage of significant findings, i.e. p-values should be identical within each column over *σ*_*C*_. However, for increasing values of *σ*_*S*_, trial-wise randomization leads to too many significant results for *σ*_*C*_ = 0 whereas subclass-wise permutation leads to a reduced number of significant findings for small values of *σ*_*C*_. (c, d) To illustrate the difference between the number of significant results when subclass variance is not present (dashed rectangle) and when it is present, we calculate normalized difference between the number of data sets with significant p-values and the number of data sets with significant p-values with the same amount of class-variance but without subclasses (significance bias, SB). This value shows that blocked permutation is unbiased for small and sufficiently large class effects and shows only a small conservative bias when subclass variance is substantially larger than class variance. Testing with trial-wise permutation, on the other hand, is too liberal when the class effect is small or null and even a small subclass effect is present. This test therefore leads to a larger number of false positive results.

To systematically study how subclasses affect significance tests, we used the simulated 100-dimensional data sets described above, produced their respective null distributions once using adjusted subclass-wise and once using trial-wise permutation tests, and calculated the expected p-values for varying sizes of class and subclass effects (Fig 5a and 5b). We used the CCR distribution of 5000 simulated data sets with no subclass- and class-effect as the null distribution for trial-wise permutation. For each individual subclass variance, we used the CCR distribution of 5000 simulated data sets with no class effect but with the respective subclass effect as the null distribution for blocked permutation. We define a measure of significance bias (SB) as the normalized difference between the number of statistically significant classification accuracy in data sets with and without subclasses. That is, $SB\;=\;P_{\sigma_{s}\neq 0}-P_{\sigma_{s}\;=\;0}$ where *P* represent the proportion of statistically significant results for a certain value of *σ*_*S*_. In contrast to false positive rates, this value can also be calculated in the presence of an effect, because data sets are simulated, and their true composition is known. Fig 5c and 5d show significance biases for block-wise and trial-wise permutation tests, respectively. Trial-wise permutation results in liberally biased p-values in the presence of subclass effects. On the other hand, block-wise permutation is slightly conservative when subclass variance is high compared with class variance. This is due to classification accuracy being a nonlinear function of class differences (here the combination of class- and subclass effects). Therefore, when the null distribution of data with subclasses is strongly biased by subclass variance, the additional influence of class variance is comparatively smaller, and the procedure will over-adjust and result in a slightly pessimistic p-value.

## Discussion

Many neuroscience data sets comprise nested subclasses, either because of requirements of the experimental design (multiple subjects, sessions, recording sites, laboratories etc.) or because of the nature of experimental stimuli [16]. We show that these subclasses can induce classifiability and systematically bias classification accuracy even when no actual class-related effect exists. This happens because subclasses have distinct centroids, particularly in high-dimensional space [2, 21]. These differences can be detected by the classifier, even when they cancel out in the classes as a whole (see Fig 1). We show empirically and analytically that classification accuracies are biased when subclasses are present in the data. We show that block-wise permutation testing on the level of subclasses can provide the correct null distribution for classification rates.

Subclasses can arise for different reasons. If subclasses originate from repeated measures, their effect can be mitigated by either using fewer repetitions and/or a higher number of different measures (stimuli, run, subjects, …). Subclasses are formed by groups of trials with a common covariance that is not related to the class difference under investigation [15, 17]. This occurs, e.g., when data are gathered in distinct blocks, when there are clusters of trials with similar physical (e.g. color) or cognitive (e.g. concepts, emotions) properties, when trials of data are correlated in time or space, or when trials belong to multiple subjects. Generally, all of these sources of subclass variance can be regarded equally. In univariate analyses, the effects of these subclasses usually cancel out and become irrelevant when subclasses are randomly distributed around the class means. Linear classifiers, however, search for combinations of linear differences that distinguish between classes. Because subclasses have non-continuous effects and are not linearly related to class membership, their influence has to be accounted for in a different way.

Two types of subclasses must be considered. If subclasses originate from repeated measures, their effect can be mitigated by either using fewer repetitions and/or a higher number of different measures (stimuli, run, subjects, …). In that case, however, the effect of subclasses cannot be entirely avoided by design and must be adjusted for during permutation testing. If subclasses pertain to other similarities between subsets of trials, then it must be considered whether these subsets actually belong to a single class or whether they should be included as distinct factors into the experimental design. For example, when I want a classifier to decide whether images of male and female faces differ, a certain classifier might distinguish stimuli by the subclass of females with long hair. Whether a successful classification based on this feature is interpreted as a genuine sex difference or as a confound related to contemporary fashion, depends on the experimenter. On the other hand, if the subclasses are induced by repeated presentation of the same faces, it is obvious that the successful above-chance classification represents a bias and does not support the hypothesis of a visible sex difference. Because this type of artefact does not occur in classical univariate testing, we believe it is important to be particularly attentive to such effects in MVPA.

Subclasses are not the same as classical confounding variables. The latter are usually continuous, normally distributed influence variables that correlate with the class variable as well as with individual features of the data. Classically, such covariates are dealt with by holding them constant across conditions (experimental control), by counterbalancing their influence (selective analysis), or by partialling out their variance through regression (statistical control). Subclasses are categorical confounds that need not correlate (share variance) with either the class variable or any individual feature. Instead, they arise from the multivariate structure introduced by shared covariance between trials. Here, in particular, we discuss nested subclasses, which occur in only one of the classes. Therefore, balancing (e.g. equal number of exemplars per category) and counterbalancing (e.g. making sure that group averages over features are equal) do not remove effects of subclasses as it would in classical statistical approaches that focus on the mean values of individual features.

The problematics of subclasses is a property that distinguishes classifiers, which can detect structure in data, from classical statistics, which concentrates on differences in means. It applies to all modalities of data, not only to EEG data, and depends on their homogeneity, which is determined by, e.g., experimental design, stimuli and subjects to be analyzed. A typical effect that could introduce subclasses in fMRI data is the presence of individual scanning runs. If each run contains only one class, the runs represent subclasses and classification accuracy will be biased.

Given a constant number of subclasses, it is the ratio between trial- and subclass-variance that determines the extent to which the within-subclass covariance affects conclusions from the data. This ratio of trial-to-subclass-variance can be quantified in terms of the intraclass correlation *ICC*. It can be shown that even a small *ICC* = 0.1 can increase the false positive rate to more than 20% when it is expected to be at α = 0.05 [16]. When MVPA is employed, the deviation of CCRs from the expected chance levels depends, aside from the number of subclasses, only on *ICC* (see Materials and methods, Appendix B), and an *ICC* as small as 0.1 can spuriously increase CCR by 10% (see Fig 3c). Given that the average *ICC*s in neuroscience is 0.19 with a range up to 0.74 [16], it can be assumed that CCRs in data with subclasses are strongly biased if the bias is not explicitly accounted for. Because parametric tests cannot account for this bias, statistical significance must be determined using the correct permutation procedure. Importantly, in a higher dimensional space, the effect introduced by subclasses accumulates over dimensions and contributes to the distinctness of the subclasses (see Fig 3).

Making subclasses less prominent in the data structure reduces the bias. According to our simulations and the analytical solution in Appendices A and B, this can be achieved in two ways: either by decreasing the subclass variance or by increasing the number of subclasses. While decreasing the subclass variance might be difficult in real-world experiments, increasing the number of subclasses is often a possibility. Although a higher number of subclasses is preferable to a lower number [14–16], six subclasses already allow reasonable permutation testing. This is because the number of possible block-wise permutation for a data set with *K* nested subclasses per class is 12(KK/2)2. Since *K* = 6 is the minimum number that exceeds 100 permutations, this number of subclasses allows to estimate p-values of up to 0.01.

The CCR is not informative about classification success when cross-validated classification is used for hypothesis testing because it does not provide a measure of statistical significance. A lower CCR might represent a more robust result, showing a higher significance level [2]. Moreover, the present simulations and experiments show that in experimental designs with nested subclasses, which are common in the life sciences, systematic dependencies in the data structure that can lead to spuriously high CCRs and null-distributions may no longer be centered on 50%. We therefore suggest that statistical significance should be tested with nonparametric permutation tests that accommodate for the bias in CCR induced by these subclasses. A more diverse range of stimuli can also be used to mitigate the bias and result in more reliable classification results. Importantly, although the results presented in the current manuscript are provided for binary classification problems, they will readily extend to multiclass case if linear classifiers are used within one-versus one or one-versus-all classification schemes [22]. This happens because such approaches use a majority voting based on single binary linear classification results, which are themselves inflated should the data set have subclasses. Nevertheless, one should note that the results of this paper are confined to the application of classification in data with nested subclasses. In data sets with crossed designs, because the subclass structure is the same within all subclasses, the subclass information can actually be used to improve classification accuracy [11, 23, 24].

Note that in the case of designs with more than one level of nested subclasses, the proposed technique in the current manuscript can still be employed. A data set with multiple levels of nested subclasses is numerically equivalent to a data set with all subclasses on one level. For instance, let’s assume that a data set, in addition to the main effect has two levels of independent, hierarchically nested subclasses. Such data can be modeled as *y*_*ijkl*_ = *C*_*i*_ + *S*_*ij*_ + *S*_*ijk*_ + *ϵ*_*ijkl*_ where *y*_*ijkl*_ are individual measurements, *C*_*i*∈[1,2]_ represents the class centroids, *S*_*ij*_ are the centroids of the *j*^*th*^ level-one subclass within class *i* and represents the vector from the class centroid to the subclass centroid, *S*_*ijk*_ are the centroids of the *k*^*th*^ level-two subclass, which are nested within subclass *S*_*ij*_, and *ϵ*_*ijkl*_ reflects the deviation from the subclass mean (*S*_*ijk*_), i.e. measurement noise. Since the sum of two normal distributions is also a normal distribution, the model can be effectively rewritten as *y*_*ij’l*_ = *C*_*i*_ + *S*_*ij'*_ + *ϵ*_*ij’l*_ where *S*_*ij'*_ = *S*_*ij*_ + *S*_*ijk*_ (with $\sigma_{S_{ij'}}^{2}\;=\;\sigma_{S_{ij}}^{2}+\sigma_{S_{ijk}}^{2}$) and j’ = {jk}, which is equivalent to the one discussed above.

An important limitation of the current considerations for linear classification is that we must assume sources of subclasses to be known. To test if data has nested subclasses in its structure, which are not known a priori or overlooked by the experimenters, clustering algorithms might be of use. First, clustering might help identifying unknown and hidden subclasses in the data. If cluster analysis finds significantly different clusters within classes, which do not correspond between classes (nested clusters), then these can be taken into account during permutation testing. Second, it is conceivable that after detecting hidden subclass clusters the cluster-related variance might be removed by subtracting cluster structure in both main classes of the training set separately. Then, for each sample of the test set, the most likely subclass is determined and the same subtraction is applied. However, it remains to be shown first that this procedure does not leak class information into the test set at some point.

It is possible to avoid subclass-related bias by proper cross-validation under some circumstances. One can use subclasses as folds during cross-validation and always leave one subclass per class out. This avoids a transfer of the learned subclass structure from training to test and will remove the corresponding bias. Note, however, that using a trial-wise permutation test will still lead to a wrong estimate of significance levels in this case. Only holding subclass items together during permutation testing results in the correct estimation of the confidence interval. In general, we recommend using split-half cross validation because of its lower variance and higher sensitivity [2] and adjusting for subclass bias during permutation testing if necessary. Both cross-validation and permutation are applied independently.

Finally, we want to mention that the suggested permutation procedure is only valid if an equal number of subclasses per class is entered into the analysis. Differences in the number of subclasses, i.e. different homogeneity of classes, can bias results. We also want to point out that linear classification is only one of the many common methods that exploit the multivariate information of a data set. Although a systematic study of subclass effects on those algorithms is beyond the scope of this paper, we believe that similar effects might be found in most classifiers that assume a continuous distribution of feature values.

## Materials and methods

### Ethics statement

This study was approved by the Ethics Committee of the Department Psychology of the LMU Munich.

### Appendix A: Theorem 1

We assume our data set consists of two sets of *N* × *K* independent random vectors ${\vec{x}}_{n}^{\left(k\right)},\;{\vec{y}}_{n^{'}}^{\left(k^{'}\right)}$ where *k*, *k*′ ∈ {1, …, *K*} labels the subclasses and *n*, *n*' ∈ {1, …, *N*}identifies the sample index in each of the subclasses. The task of the linear classifier is to separate *x* and *y* into two categories. As a model for the linear classifier, we use LDA. We therefore can map the d-dimensional vectors ${\vec{x}}_{n}^{\left(k\right)},\;{\vec{y}}_{n'}^{\left(k'\right)}$ onto the coordinates $\xi_{n}^{\left(k\right)}$ and $\eta_{n'}^{\left(k\prime \right)}\;$ w.r.t to the axis defined by the difference of the mean values of the two classes. Furthermore, we label the empirical means of the classes as:

$$\mu^{\left(k\right)}\;=\;N^{-1}\sum_{n}\xi_{n}^{\left(k\right)},\nu^{\left(k\right)}\;=\;N^{-1}\sum_{n}\eta_{n'}^{\left(k'\right)}$$

The distributions of within the subclasses is assumed to be Gaussian with variance $\sigma_{W}^{2}$, the distribution of the subclass means is also assumed to be Gaussian with variance $\sigma_{S}^{2}$ and generally different expected values $\hat{\mu },\;\hat{\nu }$. In the case of the two categories are undistinguishable the two are identical (no signal), $\hat{\mu }\;=\;\hat{\nu }$,. For every realization the means of *μ*^(*k*)^, *ν*^(*k*')^ will be different from $\hat{\mu },\hat{\nu }$, and thus we also introduce the empirical means $\overset{-}{\mu },\overset{-}{\nu }$, which underlie the estimated signal $\delta \;=\;\overset{-}{\mu }-\overset{-}{\nu }$.

Besides, we can compute the total variance of the data set as:

$$\begin{array}{l} \sigma^{2}=\frac{1}{2}\left[var\left(\xi \right)+var\left(\eta \right)\right]=var\left(\xi \right)=<{\left[\xi -\hat{\mu }\right]}^{2}>\\ =\;<{\left[\left(\xi -\mu^{\left(k\right)}\right)+\left(\mu^{\left(k\right)}-\hat{\mu }\right)\right]}^{2}>\;=\;\sigma_{w}^{2}+\;\sigma_{s}^{2} \end{array}$$

Under these conditions, assuming that the total variance *σ*^2^ is constant, the expected CCR for such data can be estimated as:

$$CCR\;=\;\int_{-\infty }^{+\infty }dq\frac{sign((1+\frac{\rho }{K})q+\tilde{\delta })}{2}N(q)\left[erf(\frac{q\frac{\rho }{K}+\frac{\tilde{\delta }}{2}}{\sqrt{2}\lambda })+erf(\frac{q+\frac{\tilde{\delta }}{2}}{\sqrt{2}\lambda })\right]$$

With *N*(*q*) denoting the normal distribution,$\;\rho \;=\;\frac{\sigma_{s}^{2}}{\sigma^{2}}$,$\;\tilde{\delta }\;=\;\frac{\delta }{\sigma }\sqrt{1+\frac{\rho }{K}}$, and $\lambda^{2}\;=\;\frac{\rho }{2K}(1-\frac{\rho }{K})$.

#### Proof

For LDA, the Correct Classification Rate (CCR) can be computed as the probability that during testing, a data point of class *x* is on the same side of the classification threshold $\theta \;=\;\frac{\overset{-}{\mu }+\overset{-}{\nu }}{2}$ as the empirical mean $\hat{\mu }$, and a data point of class *y* is on the opposite side:

$$CCR=\left[p\left(\xi >\theta ,\bar{\mu }>\bar{\nu }\right)+p\left(\xi <\theta ,\bar{\mu }<\bar{\nu }\right)\right]p\xi +\left[p\left(\eta >\theta ,\bar{\mu }>\bar{\nu }\right)+p\left(\eta <\theta ,\bar{\mu }<\bar{\nu }\right)\right]p\eta$$ (2)

Under the assumption of symmetry between class labels, i.e., $p\xi \;=\;p\eta \;=\;\frac{1}{2}$, equally distributed subclass means, and equally within-class distributions Eq (2) must be symmetrical with respect to exchanging *x* and *y* and thus we can obtain the CCR from

$$CR=p\left(\xi >\theta ,\bar{\mu }>\bar{\nu }\right)+p\left(\xi <\theta ,\bar{\mu }<\bar{\nu }\right)$$ (3)

Denoting $\vec{\mu }\;=\;{\left(\mu^{\left(1\right)},\ldots ,\mu^{\left(K\right)}\right)}^{T}$ and $\vec{\nu }\;=\;{\left(\nu^{\left(1\right)},\ldots ,\nu^{\left(K\right)}\right)}^{T}$, we thus can write

$$\begin{array}{c} CCR=\;\int d\vec{\mu }d\vec{\nu }p\left(\xi >\theta ,\bar{\mu }>\bar{\nu |}\vec{\mu },\vec{\nu }\right)p\left(\vec{\mu },\vec{\nu }\right)+\;p\left(\xi <\theta ,\bar{\mu }<\bar{\nu |}\vec{\mu },\vec{\nu }\right)p\left(\vec{\mu },\vec{\nu }\right)\\ =\;\int d\vec{\mu }d\vec{\nu }p\left(\vec{\mu },\vec{\nu }\right)\left[\int_{\theta }^{\infty }d\xi p\left(\xi |\vec{\mu },\vec{\nu }\right)Η\left(\bar{\mu }-\bar{\nu }\right)+\;\int_{-\infty }^{\theta }d\xi p\left(\xi |\vec{\mu },\vec{\nu }\right)Η\left(\bar{\nu }-\bar{\mu }\right)\right] \end{array}$$ (4)

with *H* denoting the Heaviside step function. Substituing *ξ* = *θ* + *t*, the integrals of *ξ* can be transformed to

$$CCR=\;\int_{0}^{\infty }dt\int d\vec{\mu }d\vec{\nu }p\left(\vec{\mu },\vec{\nu }\right)\times \left[p\left(t+\theta |\vec{\mu },\vec{\nu }\right)Η\left(\bar{\mu }-\bar{\nu }\right)+p\left(-t+\theta |\vec{\mu },\vec{\nu }\right)Η\left(\bar{\nu }-\bar{\mu }\right)\right]$$ (5)

The subsample means are independent $\;p(\vec{\mu },\vec{\nu })\;=\;\prod_{k}p(\mu^{\left(k\right)})\prod_{k'}p(\nu^{\left(k'\right)})$. Moreover, $\vec{\nu \;}$ only affects the integral with its mean $\overset{-}{\mu }$, and thus $d\vec{\nu }p(\vec{\nu })\;=\;d\overset{-}{\nu }p(\overset{-}{\nu }).$

All distributions are assumed to be Gaussians. We therefore can express all probabilities by Gaussian distribution *G*. In particular

$$\begin{array}{cc} p(\xi \vec{\mu },\vec{\nu }) & =\;\frac{1}{K}\sum_{k}G(\xi -\mu^{\left(k\right)},\sigma_{w})\\ p\left(\bar{\nu }\right) & =G\left(\bar{\nu }-\hat{\nu },\sigma_{s}/\sqrt{K}\right)\\ p\left(\mu^{\left(k\right)}\right) & =G\left(\mu^{\left(k\right)}-\hat{\mu },\sigma_{s}\right) \end{array}$$ (6)

Inserting Eq (6) into Eq (5), we obtain:

$$\begin{array}{c} CCR=\frac{1}{K}\sum_{k}\int_{0}^{\infty }dt\left(\prod_{k}d\mu^{\left(k\right)}G\left(\mu^{\left(k\right)}-\hat{\mu },\sigma_{s}\right)\right)\\ \times [\int_{-\infty }^{\bar{\mu }}d\bar{\nu }G\left(\bar{\nu }-\hat{\nu },\sigma_{s}/\sqrt{K}\right)G\left(t+\theta -\mu^{\left(k\right)},\sigma_{w}\right)\\ +\int_{\bar{\mu }}^{\infty }d\bar{\nu }G\left(\bar{\nu }-\hat{\nu },\sigma_{s}/\sqrt{K}\right)G\left(-t+\theta -\mu^{\left(k\right)},\sigma_{w}\right)] \end{array}$$ (7)

Substituting $u\;=\;\overset{-}{\nu }-\overset{-}{\mu }\;$ the integrals over $\overset{-}{\nu }\;$ can be combined such that

$$\begin{array}{c} CCR=\frac{1}{K}\sum_{k}\int_{0}^{\infty }dt\int_{0}^{\infty }du\left(\prod_{k}d\mu^{\left(k\right)}G\left(\mu^{\left(k\right)}-\hat{\mu },\sigma_{s}\right)\right)\\ \times [G\left(\bar{\mu }-\hat{\nu }-u,\sigma_{s}/\sqrt{K}\right)G\left(\bar{\mu }-\mu^{\left(k\right)}+t-u/2,\sigma_{w}\right)\\ +G\left(\bar{\mu }-\hat{\nu }-u,\sigma_{s}/\sqrt{K}\right)G\left(\bar{\mu }-\mu^{\left(k\right)}-t+u/2,\sigma_{w}\right)] \end{array}$$ (8)

Completing squares in the last two Gaussian distributions and integrating over all *μ*^(*k*)^ with ϗ≠k, yields

$$\begin{array}{c} CCR=\frac{1}{K}\sum_{k}\int_{0}^{\infty }dt\int_{0}^{\infty }du\int d\mu^{\left(k\right)}G(\mu^{\left(k\right)}-\hat{\mu },\sigma_{s})\\ \times K[G\left(\mu^{\left(k\right)}\left(1-K{\left(\frac{\sigma_{s}}{\sigma^{*}}\right)}^{2}\right)+\left(K-1\right)\hat{\mu }+\tilde{C}\left(u,t\right),\sum *\right)\times G\left(\mu^{\left(k\right)}-B_{+}\left(u,t\right),\sigma^{*}/\sqrt{K}\right)\\ +K\left[G\left(\mu^{\left(k\right)}\left(1-K{\left(\frac{\sigma_{s}}{\sigma^{*}}\right)}^{2}\right)+\left(K-1\right)\hat{\mu }+{\tilde{C}}_{-}\left(u,t\right),\sum *\right)\times G\left(\mu^{\left(k\right)}-B_{-}\left(u,t\right),\sigma^{*}/\sqrt{K}\right)\right] \end{array}$$ (9)

With ${\left(\sigma^{*}\right)}^{2}\;=\;K\sigma_{w}^{2}+\sigma_{s}^{2},\;{\left({Ʃ}^{*}\right)}^{2}\;=\;(K-1)\sigma_{s}^{2}+{\left(K\sigma_{w}\sigma_{s}/\sigma^{*}\right)}^{2},\;B_{\pm }(u,t)\;=\;\hat{\nu }\pm (t+u/2)$,

And

$$\tilde{C_{\pm }}(u,t)\;=\;(\pm K(t-\frac{u}{2})\sigma_{s}^{2}-K^{2}(\hat{\nu }\pm u)\sigma_{w}^{2})/{\left(\sigma^{*}\right)}^{2}$$

Again, completing squares of the first and third Gaussian distribution results in

$$G(\mu^{\left(k\right)}-\hat{\mu },\sigma_{s})G(\mu^{\left(k\right)}-B_{\pm }(u,t),\frac{\sigma^{*}}{\sqrt{K}})\;=\;G(\hat{\mu }-B_{+}(u,t),\sigma^{**}/\sqrt{K})\times G(\mu^{\left(k\right)}-D_{\pm }(u,t),\frac{\sigma^{*}\sigma_{s}}{\sigma^{**}})$$

With ${\left(\sigma^{**}\right)}^{2}\;=\;K\sigma_{s}^{2}+{\left(\sigma^{*}\right)}^{2}$ and

$$D_{\pm }(u,t)\;=\;\hat{\mu }{\left(\frac{\sigma^{*}}{\sigma^{**}}\right)}^{2}+KB_{\pm }(u,t){\left(\frac{\sigma_{s}}{\sigma^{**}}\right)}^{2}$$

Solving the integral over *μ*^(*k*)^ in Eq (9) as a convolution of two Gaussians, we end up at

$$\begin{array}{c} CCR=\int_{0}^{\infty }dt\int_{0}^{\infty }du[G\left(\hat{\mu }-\hat{\nu }-\left(t+u/2\right),\sigma^{**}/\sqrt{K}\right)\\ \times G\left(\alpha \left(\hat{\mu }-\hat{\nu }\right)-\beta u+\gamma t,\sum **/\sqrt{K}\right)\\ +G\left(\hat{\mu }-\hat{\nu }+\left(t+u/2\right),\sigma^{**}/\sqrt{K}\right)\times G\left(\alpha \left(\hat{\mu }-\hat{\nu }\right)+\beta u+\gamma t,\sum **/\sqrt{K}\right)] \end{array}$$ (10)

With $\alpha \;=\;1-2{\left(\frac{\sigma_{s}}{\sigma^{**}}\right)}^{2}$,$\;\beta \;=\;1-{\left(\frac{\sigma_{s}}{\sigma^{**}}\right)}^{2},\;\gamma \;=\;2{\left(\frac{\sigma_{s}}{\sigma^{**}}\right)}^{2}$, and

$${\left({Ʃ}^{**}\right)}^{2}\;=\;{\left({Ʃ}^{*}\right)}^{2}+{\left((1-K{\left(\frac{\sigma_{s}}{\sigma^{*}}\right)}^{2})\frac{\sigma^{*}\sigma_{s}}{\sigma^{**}}\right)}^{2}$$

Introducing the signal $\delta \;=\;\hat{\mu }-\hat{\nu }\;$ and substituting *ν* = *t* + *u*/2 ∓ *δ* yields

$$\begin{array}{c} CCR=2\int_{-\infty }^{\infty }dt\int_{-\infty }^{\infty }d\nu G\left(\nu ,\sigma^{**}/\sqrt{K}\right)\\ \times [H\left(\nu -t+\delta \right)G\left(t-\beta \nu -\delta /2,\sum^{**}/\left(2K\right)\right)\\ +H\left(\nu -t-\delta \right)G\left(t-\beta \nu +\delta /2,\sum^{**}/\left(2K\right)\right)]\frac{1}{2}\\ =\int_{\infty }^{+\infty }d\nu \frac{sign\left(\nu +\delta \right)}{2}G\left(\nu ,\sigma^{**}/\sqrt{K}\right)\left[erf\left(\frac{\gamma \nu +\delta }{\sqrt{2\sum^{**}/K}}\right)+erf\left(\frac{\left(2-\gamma \right)\nu +\delta }{\sqrt{2\sum^{**}/K}}\right)\right]\\ =\int_{\infty }^{+\infty }dq\frac{sign\left(\left(1+\frac{\rho }{K}\right)q+\tilde{\delta }\right)}{2}N\left(q\right)\left[erf\left(\frac{q\frac{\rho }{K}+\frac{\tilde{\delta }}{2}}{\sqrt{2}\lambda }\right)+erf\left(\frac{q+\frac{\tilde{\delta }}{2}}{\sqrt{2}\lambda }\right)\right] \end{array}$$ (11)

With *N*(*q*) denoting the normal distribution,$\;\rho \;=\;\frac{\sigma_{s}^{2}}{\sigma^{2}}$,$\;\tilde{\delta }\;=\;\frac{\delta }{\sigma }\sqrt{1+\frac{\rho }{K}}$, and $\lambda^{2}\;=\;\frac{\rho }{2K}(1-\frac{\rho }{K})$.

### Appendix B: Corollary 1

Classification accuracy for data sets with no main effect $\left(\hat{\mu }\;=\;\hat{\nu }\right)$ depends only on number of subclasses and intraclass correlation *ICC* and can be estimated by $\;CCR\;=\;1-\frac{1}{\pi }arctan(\sqrt{2(\frac{K}{ICC}-1)})$.

#### Proof

Substituting for Eq 11 with *δ* = 0 and noting that *ICC* = *ρ* we have

$$\begin{array}{cc} CCR & =\;\int_{-\infty }^{+\infty }dq\frac{sign\left(\left(1+\frac{\rho }{K}\right)q\right)}{2}N\left(q\right)[erf\left(\frac{q\frac{\rho }{K}}{\sqrt{2}\lambda }\right)+erf\left(\frac{q}{\sqrt{2}\lambda }\right)\\ & =\int_{-\infty }^{+\infty }dq\frac{sign\left(q\right)}{2}N\left(q\right)\left[erf\left(\frac{q\frac{\rho }{K}}{\sqrt{2}\lambda }\right)+erf\left(\frac{q}{\sqrt{2}\lambda }\right)\right] \end{array}$$

Noting that *sign*(*q*), *erf*(*q*) are odd functions and *N*(*q*) is and even function of *q*, we have

$$\begin{array}{c} CCR=\frac{1}{2}\overset{+\infty }{\underset{0}{\int }}dqN\left(q\right)\left[erf\left(\frac{q\frac{\rho }{K}}{\sqrt{2}\lambda }\right)+erf\left(\frac{q}{\sqrt{2}\lambda }\right)\right]\\ =\frac{1}{2}\overset{+\infty }{\underset{0}{\int }}dqN\left(q\right)erf\left(\frac{q\frac{\rho }{K}}{\sqrt{2}\lambda }\right)+\frac{1}{2}\overset{+\infty }{\underset{0}{\int }}dqN\left(q\right)erf\left(\frac{q}{\sqrt{2}\lambda }\right)\\ =\frac{1}{2}-\frac{1}{\pi }arctan\left(\lambda \right)+\frac{1}{2}-\frac{1}{\pi }arctan\left(\frac{\lambda K}{\rho }\right)\\ =1-\frac{1}{\pi }\left(arctan\left(\lambda \right)+arctan\left(\frac{\lambda K}{\rho }\right)\right)=1-\frac{1}{\pi }arctan\left(\sqrt{2\left(\frac{K}{\rho }-1\right)}\right) \end{array}$$ (12)

Note that the closed form of CCR in data sets with no effect size depend only on *ICC* = *ρ* and *K*.

Note that the expected correct classification using LDA for data sets with no effect ($\hat{\mu }\;=\;\hat{\nu }$) is an increasing function of subclass variance $\sigma_{S}^{2}$, a decreasing function of number of subclasses *K*, and is 50% only when *ICC* = 0.

## References

[1] Haxby JV, Connolly AC, Guntupalli JS. Decoding Neural Representational Spaces Using Multivariate Pattern Analysis. Annu Rev Neurosci. 2014;37:435–456. 10.1146/annurev-neuro-062012-170325 25002277

[2] Jamalabadi H, Alizadeh S, Schönauer M, Leibold C, Gais S. Classification based hypothesis testing in neuroscience: Below-chance level classification rates and overlooked statistical properties of linear parametric classifiers. Hum Brain Mapp. 2016;37(5):1842–1855. 10.1002/hbm.23140 27015748 PMC6867424

[3] Stelzer J, Chen Y, Turner R. Statistical inference and multiple testing correction in classification-based multi-voxel pattern analysis (MVPA): random permutations and cluster size control. NeuroImage. 2013;65:69–82. 10.1016/j.neuroimage.2012.09.063 23041526

[4] Guyon I, Weston J, Barnhill S, Vapnik V. Gene selection for cancer classification using support vector machines. Mach Learn. 2002;46(1–3):389–422.

[5] Brown MPS, Grundy WN, Lin D, Cristianini N, Sugnet CW, Furey TS, et al Knowledge-based analysis of microarray gene expression data by using support vector machines. P Natl Acad Sci USA. 2000;97(1):262–267. 10.1073/pnas.97.1.262 PMC26651 10618406

[6] Zien A, Ratsch G, Mika S, Scholkopf B, Lengauer T, Muller KR. Engineering support vector machine kernels that recognize translation initiation sites. Bioinformatics. 2000;16(9):799–807. 11108702 10.1093/bioinformatics/16.9.799

[7] Woolgar A, Golland P, Bode S. Coping with confounds in multivoxel pattern analysis: what should we do about reaction time differences? A comment on Todd, Nystrom & Cohen 2013. NeuroImage. 2014;98:506–512. 10.1016/j.neuroimage.2014.04.059 24793832 PMC4450346

[8] Haynes JD. A Primer on Pattern-Based Approaches to fMRI: Principles, Pitfalls, and Perspectives. Neuron. 2015;87(2):257–270. 10.1016/j.neuron.2015.05.025 26182413

[9] Norman KA, Polyn SM, Detre GJ, Haxby JV. Beyond mind-reading: multi-voxel pattern analysis of fMRI data. Trends Cogn Sci 2006;10(9):424–430. 10.1016/j.tics.2006.07.005 16899397

[10] Alizadeh S, Jamalabadi H, Schönauer M, Leibold C, Gais S. Decoding cognitive concepts from neuroimaging data using multivariate pattern analysis. Neuroimage. 2017;159:449–458. 10.1016/j.neuroimage.2017.07.058 28765057

[11] Hohne J, Bartz D, Hebart MN, Muller KR, Blankertz B. Analyzing neuroimaging data with subclasses: A shrinkage approach. Neuroimage. 2016;124(Pt A):740–751. 10.1016/j.neuroimage.2015.09.031 26407815

[12] Hebart MN, Baker CI. Deconstructing multivariate decoding for the study of brain function. Neuroimage. 2017. 10.1016/j.neuroimage.2017.08.005 PMC5797513 28782682

[13] Galbraith S, Daniel JA, Vissel B. A study of clustered data and approaches to its analysis. J Neurosci Methods. 2010;30(32):10601–10608. 10.1523/JNEUROSCI.0362-10.2010 PMC6634702 20702692

[14] Anderson MJ, Ter Braak CJF. Permutation tests for multi-factorial analysis of variance. J Stat Comput Sim. 2003;73(2):85–113.

[15] Lazic SE. The problem of pseudoreplication in neuroscientific studies: is it affecting your analysis? BMC Neurosci. 2010;11:5 10.1186/1471-2202-11-5 20074371 PMC2817684

[16] Aarts E, Verhage M, Veenvliet JV, Dolan CV, van der Sluis S. A solution to dependency: using multilevel analysis to accommodate nested data. Nat Neurosci. 2014;17(4):491–496. 10.1038/nn.3648 24671065

[17] Todd MT, Nystrom LE, Cohen JD. Confounds in multivariate pattern analysis: Theory and rule representation case study. NeuroImage. 2013;77:157–165. 10.1016/j.neuroimage.2013.03.039 23558095

[18] Malone PS, Glezer LS, Kim J, Jiang X, Riesenhuber M. Multivariate Pattern Analysis Reveals Category-Related Organization of Semantic Representations in Anterior Temporal Cortex. J Neurosci. 2016;36(39):10089–10096. 10.1523/JNEUROSCI.1599-16.2016 27683905 PMC6705575

[19] Delorme A, Makeig S. EEGLAB: an open source toolbox for analysis of single-trial EEG dynamics including independent component analysis. J Neurosci Methods. 2004;134(1):9–21. 10.1016/j.jneumeth.2003.10.009 15102499

[20] Winkler AM, Webster MA, Vidaurre D, Nichols TE, Smith SM. Multi-level block permutation. Neuroimage. 2015;123:253–268. 10.1016/j.neuroimage.2015.05.092 26074200 PMC4644991

[21] Fan JQ, Fan YY. High Dimensional Classification Using Features Annealed Independence Rules. Ann Stat. 2008;36(6):2605–2637. 10.1214/07-AOS504 19169416 PMC2630123

[22] Tax DM, Duin RP: Using two-class classifiers for multiclass classification. In: Proceedings of 16th International Conference on Pattern Recognition. IEEE; 2002. pp. 124–127.

[23] Hastie T, Tibshirani R. Discriminant analysis by Gaussian mixtures. J Roy Stat Soc B Met. 1996;58(1):155–176.

[24] Zhu M, Martinez AM. Subclass discriminant analysis. IEEE Trans Pattern Anal Mach Intell. 2006;28(8):1274–1286. 10.1109/TPAMI.2006.172 16886863

